# Successful direct intrahepatic portosystemic shunt (DIPS) creation following transmesenteric porta hepatis access in a young patient with recurrent variceal bleeding

**DOI:** 10.1186/s42155-023-00377-8

**Published:** 2023-12-19

**Authors:** Mari Tanaka, Kei Yamada, Sanjeeva Kalva

**Affiliations:** 1 Massachusetts General Interventional Radiology, Massachusetts General Hospital, 55 Fruit Street, Boston, MA 02114 USA; 2 Harvard Medical School, 22 Shattuck Street, Boston, MA 02115 USA

**Keywords:** Portal hypertension, Intracardiac US ICE, TIPS/DIPS

## Abstract

**Background:**

Transmesenteric access for portal vein reconstruction and transjugular intrahepatic portosystemic shunt allows for intervention in patients with unfavorable anatomy and can be performed via multiple methods but may be difficult to obtain in patients with complex anatomy.

**Case presentation:**

We present a case report describing a method of obtaining transmesenteric access in the porta hepatis to facilitate direct intrahepatic portosystemic shunt creation in a young patient with recurrent variceal bleeding. This patient anatomy was unfavorable, and initially he was thought to be a poor candidate for any intervention, but this technique allowed for successful decompression of the varices safely and effectively.

**Conclusions:**

This is a technique to consider in similar complex cases and expands treatment for those who previously would not have been considered for intrahepatic shunt formation.

**Graphical Abstract:**

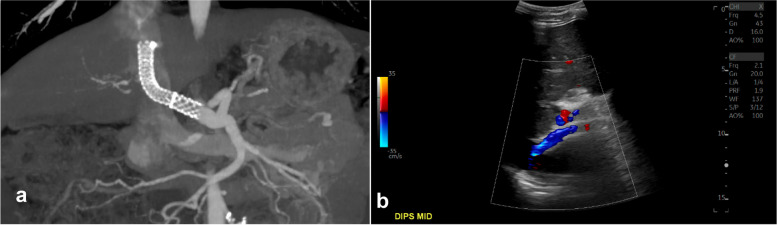

**Supplementary Information:**

The online version contains supplementary material available at 10.1186/s42155-023-00377-8.

## Background

Transmesenteric access for portal vein reconstruction and transjugular intrahepatic portosystemic shunt (PVR-TIPS) for chronic portal vein thrombus (PVT) is an option for patients with anatomy that precludes transsplenic access. Previous techniques have described ultrasound (US), computed tomography (CT) guided access, and even surgical exposure of the superior mesenteric vein (SMV) or inferior mesenteric vein (IMV) [1–4].

This report describes a complex case where US-guided attempt to access a SMV collateral in the porta hepatis for PVR-TIPS resulted in advertent replaced right hepatic artery (RHA) puncture, which guided a fluoroscopic access to the SMV collateral and successful direct intrahepatic portosystemic shunt (DIPS) creation.

## Case presentation

A 35-year-old man was referred to our division with concern of recurrent duodenal variceal bleeding secondary to portal hypertension from chronic PVT since infancy from Factor V Leiden deficiency. His vital signs were within normal limits and laboratory values unremarkable. His history was notable for numerous failed surgical portal-systemic shunts, splenectomy, multiple upper endoscopies with banding of varices, and an endoscopic glue-embolization of duodenal varices that resulted in renal infarction. He was medically managed on Nadolol but given multiple episodes of bleeding and lack of surgical options, he was referred to interventional radiology (IR). The patient was seen in IR clinic where PVR-TIPS and DIPS in addition to their risks and benefits were both discussed with him. Risks discussed ranged from bleeding, infection, hepatic encephalopathy, liver failure, inability to perform the procedure to death given the anticipated complexity of the procedure. Given his lack of other treatment options and the concern for recurrent life-threatening bleeding, the benefits of the procedure were felt to outweigh the risks, and the patient opted to proceed.

**Fig. 1:**
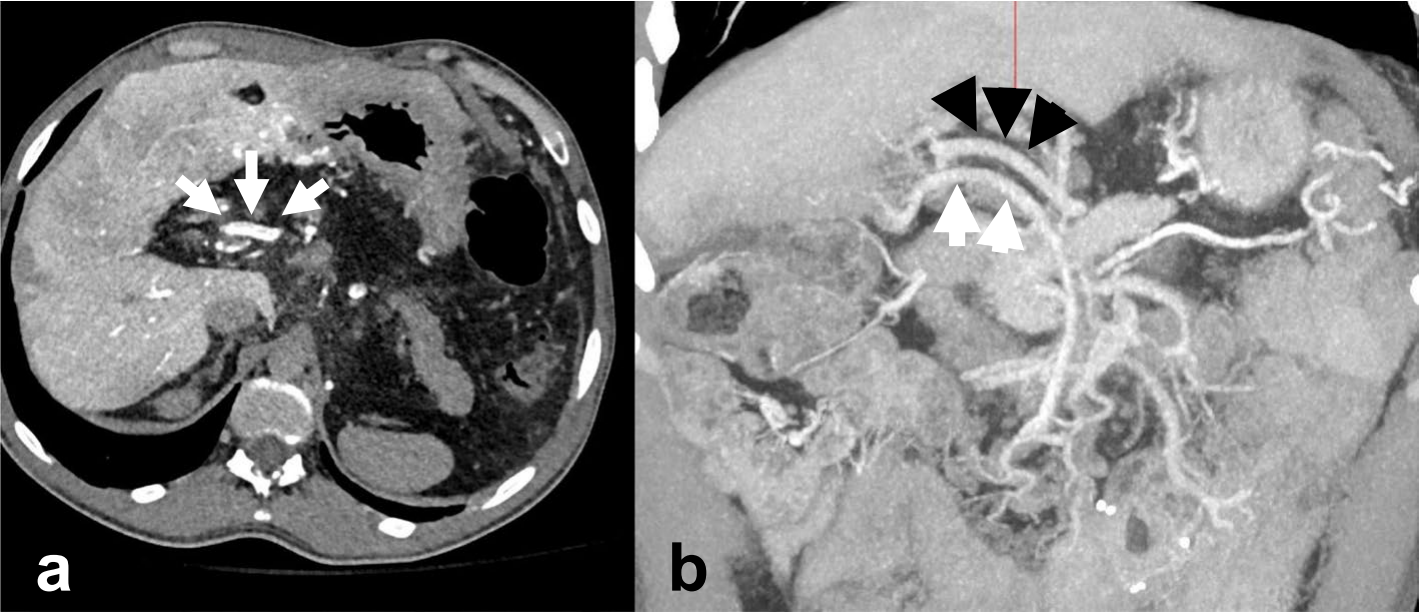
A CT arterioportography axial image with contrast injected via an SMA catheter showed (**a**) known chronic portal vein thrombus and prominent SMV collateral in the porta hepatis (white arrows). A coronal image from multiplanar reformatted maximum intensity projections shows the relationship of the HA (black arrows) relative to the dominant collateral (white arrows)

Given the complex post-surgical anatomy and long-standing thrombus, a CT arterioportography was performed with an arterial catheter positioned in the SMA. This demonstrated chronic PVT, absent splenic vein, and a patent SMV with prominent collateral in the porta hepatis (Fig. 1a, b), which ran parallel to the prominent replaced right hepatic artery (RHA) arising from the SMA. Numerous abdominal varices were noted and reconstituted the intrahepatic portal veins (PV). Additionally, patient had chronic biliary ductal dilation, unchanged from prior imaging.

A small possible channel between the hepatic veins and dominant portal collateral to target for a PVR-TIPS was seen on imaging. Given the presence of this channel and patient’s young age, the decision was made to attempt PVR-TIPS first despite low-likelihood of success to fully exonerate it as an option, prior to pursuing direct intrahepatic portosystemic shunt (DIPS) placement.

**Fig. 2:**
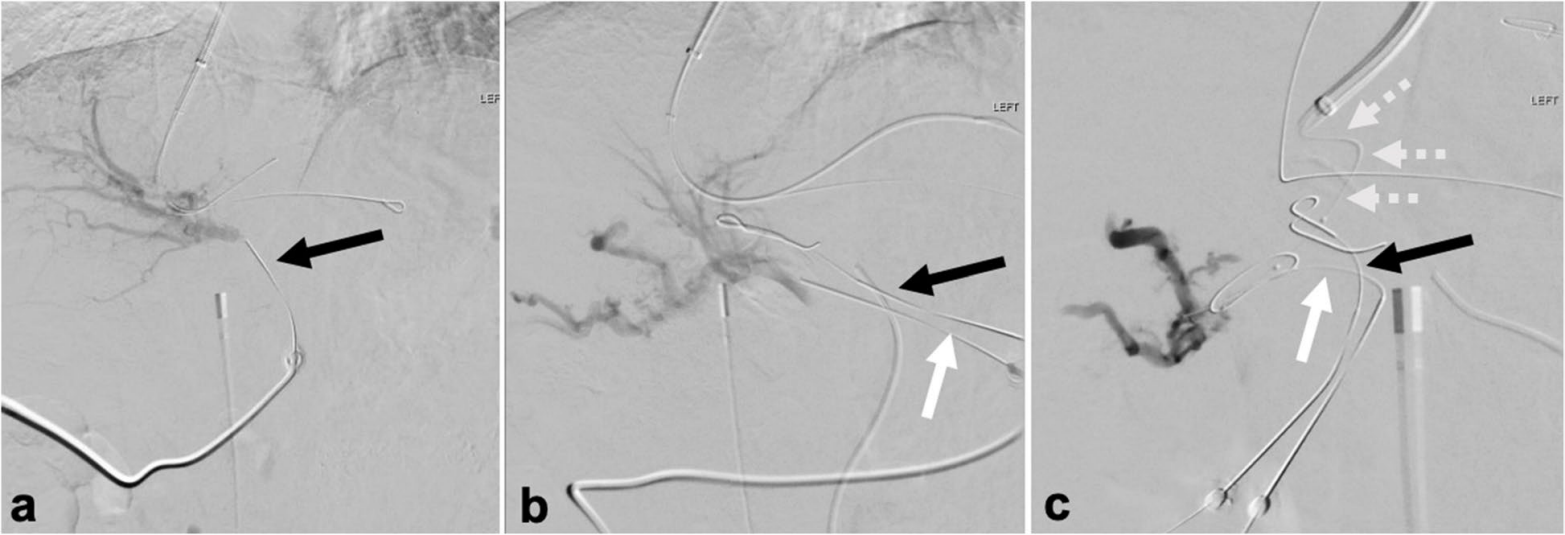
Digital subtraction images from procedure show (**a**) contrast in the hepatic artery via a 21G needle placed under ultrasound access (black arrow). **b** This access was used as a landmark to place a second 21G needle under fluoroscopic guidance into the portal system confirmed with contrast injection. **c** A 21G needle was used under fluoroscopy to puncture the SMV collateral from the IVC and a 1.5F support catheter (white dashed arrows) was navigated across the parenchymal tract into the portal system confirmed via contrast injection

Under intracardiac echo (ICE) guidance, a 16G transjugular needle (Ring set; Cook Medical, Bloomington, Indiana) was used to access the portal system from the middle hepatic vein. Attempts to access the extrahepatic portal vein via a small possible channel seen on planning images were unsuccessful, so decision was made to percutaneously access the SMV collateral in the porta hepatis. A 21-gauge needle (AccuStick System; Boston Scientific, Marlborough, Massachusetts) was placed under US into a vascular structure at the porta hepatis favored to represent the target collateral. Visualization was challenging and complex due to numerous enlarged echogenic structures from biliary ductal dilation, hypertrophied replaced right hepatic artery, multiple additional venous collaterals. Injection of contrast material via this access opacified the replaced RHA (Fig. 2a). Superior mesenteric angiogram via a right common femoral artery access demonstrated no evidence of active extravasation or significant arterial injury. This arterial access provided a fluoroscopic landmark for placing a second 21-gauge needle into the target SMV collateral noted to be inferior to the artery (Fig. 2b).

**Fig. 3:**
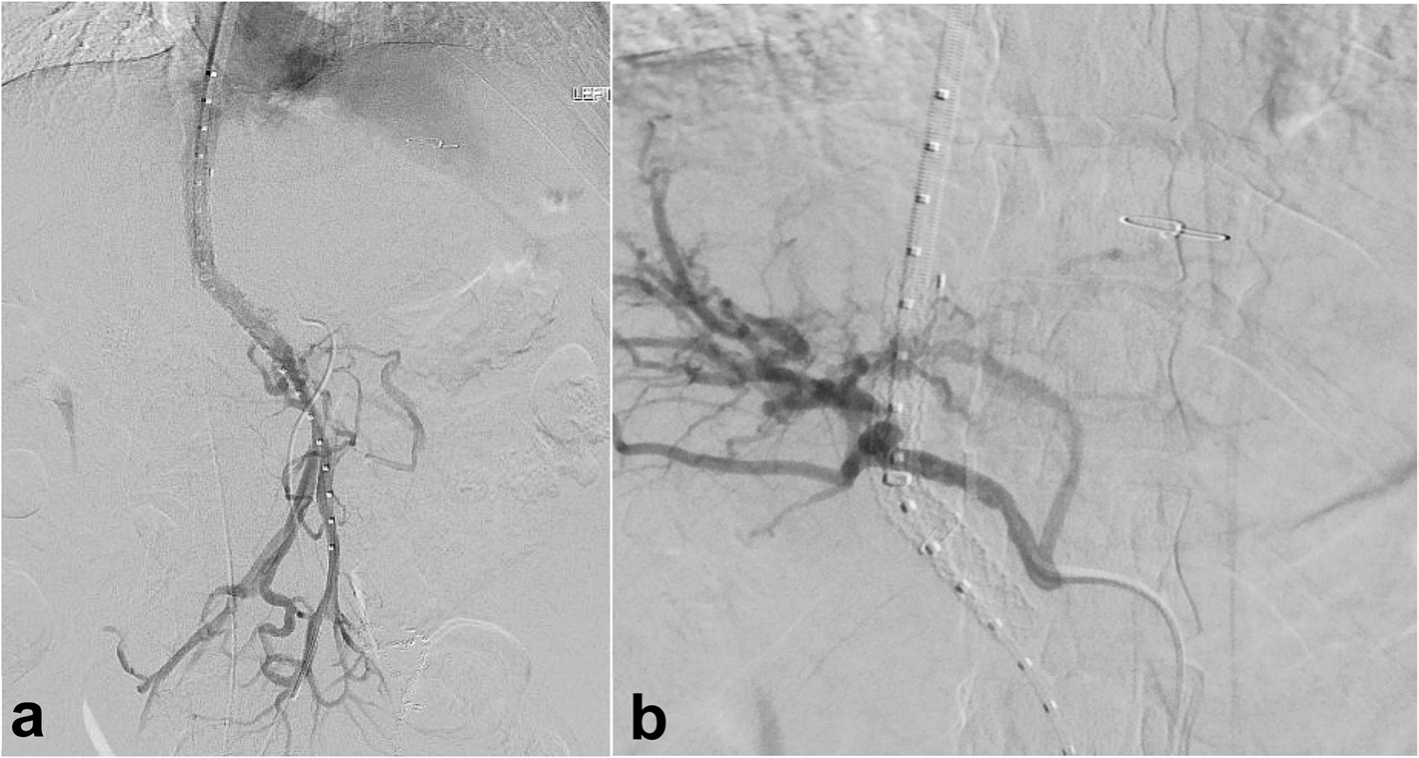
Digital subtraction angiogram images from the procedure show (**a**) widely patent DIPS stent and (**b**) no pseudoaneurysm or significant dissection in the right hepatic artery via transfemoral arterial catheter in the RHA following removal of percutaneous wire and needle

Given the visualized hepatic arterial anatomy and concern a TIPS stent would traverse prominent multiple arterial branches, decision was made to pursue a DIPS between the accessed collateral and the IVC. Visualization of target collateral via ICE in the IVC was suboptimal and given the presence of fluoroscopic targets from the percutaneous access needles and wires, decision was made to perform transhepatic access fluoroscopically. A 65 cm 21-gauge needle (Chiba; Cook Medical, Bloomington, Indiana) was used under fluoroscopy to puncture the SMV collateral from the IVC, and traversing the liver parenchyma was difficult and required numerous catheters and wires. Eventually, a 1.5F support catheter (Quick-Cross; Philips, Amsterdam, Netherlands) was navigated successfully into the SMV collateral, which was confirmed on digital subtraction angiogram (Fig. 2c). The intrahepatic tract was dilated, and an 8–10 mm × 6/2 cm endoprosthesis (VIATORR; Gore, Flagstaff, Arizona) was placed, and post-dilated to 10 mm. Post-stent venography demonstrated flow through the DIPS, but thrombus in the stent and SMV. Suction thrombectomy (Indigo Lighting CAT12; Penumbra, Alameda, California) was performed and resulted in improved flow and thrombus resolution (Fig. 3a). A repeat RHA angiogram with 21G needle in place demonstrated no evidence of injury, so the needle was removed, and angiograms repeated in multiple obliques was negative for extravasation or significant arterial injury (Fig. 3b).

The patient tolerated the procedure well and was admitted overnight for observation. He was started on a heparin drip and transitioned to Apixaban at discharge on post-procedure day 1. At his 1-month follow-up visit, he felt well without recurrent bleeding. An abdominal US and CT demonstrated a widely patent DIPS and no injury to the RHA at that time. At 7-months post-procedure, the patient continues to feel well without hepatic encephalopathy or recurrent bleeding. US and CT imaging demonstrated a widely patent DIPS (Supplemental Fig. 1a, b) He will continue to be closely followed with US imaging, IR clinic visits, and close gastroenterology follow-up.

## Conclusions

Transmesenteric access via US and CT for PVR-TIPS in patients where the splenic vein is not accessible has been described [2, 4]. A small series of 4 patients where transmesenteric US access was used for PVR-TIPS described 100% technical success, TIPS patency at 1 month, and no complications [3]. Our patient had anatomy such that access of the SMV and IMV was not feasible and the target SMV collateral in the porta hepatis put the large RHA at risk for puncture. While this access allowed for a target for portal access, it also allowed for visualization of the prominent RHA branches that resulted in abandoning a TIPS in favor of DIPS to reduce the risk of further arterial injury. It is possible that the low incidence of hepatic artery injury of < 1% described in the literature is an underestimate due to compensation mechanisms such that hepatic artery injuries are not clinically noted and that we were biased by their visualization which is rarely performed in such cases [5]. However, given the size of the adjacent vessels and desire to reduce further risk of injury given more proximal hepatic artery access and potential injury, we felt it better to place the DIPS.

DIPS creation using intra-vascular ultrasound (IVUS) has been previously described as a safe and effective method for DIPS creation [6]. This technique targets puncturing from the intrahepatic IVC to the intrahepatic main portal vein spanning from its bifurcation in to right and left portal vein to 2 cm caudal from the bifurcation, but visualization can be difficult based on anatomy and depth. This can result in extrahepatic portal access, which resulted in bleeding complications in the two patients in which extrahepatic portal access occurred [6]. Our patient had neither intrahepatic portal veins as a viable target, nor anatomy that allowed for good visualization to allow for a safe IVUS-guided access.

Use of selective wire cannulation of a hepatic artery branch as guidance for TIPS has been described as a safe and effective method for transhepatic access instead of CO2 or ICE guidance [7].

Transmesenteric access while helpful for many patients, can still be challenging in patients with complex anatomy. Learning points from this case include understanding that access in the porta hepatis increases risk of hepatic arterial injury, which is otherwise rare in conventional TIPS (< 1%) [5]. Hepatic arterial access in this case, while not planned, allowed for safe DIPS resulting in a good outcome for a young patient previously labeled as an “impossible TIPS” candidate. Knowledge of alternative techniques and advanced adjunctive maneuvers to employ during TIPS or DIPS creation is of importance as patients may have complex or challenging anatomy not conducive to conventional techniques.

### Supplementary Information

**Additional file 1:** **Supplemental Figure 1.** A coronal image from multiplanar reformatted maximum intensity projections created from a CT angiogram performed 1-month post-procedure (a) demonstrates patency of DIPS stent and a subsequent (b) US performed 2-months post procedure demonstrate stent patency.

## Data Availability

Data sharing is not applicable to this article as no datasets were generated or analyzed during the current study.

## Abbreviations

PVR-TIPS: Portal vein reconstruction and transjugular intrahepatic portosystemic shunt
PVT: Portal vein thrombus
US: Ultrasound
CT: Computed Tomography
SMV: Superior mesenteric vein
IMV: Inferior mesenteric vein
RHA: Right hepatic artery
DIPS: Direct intrahepatic portosystemic shunt
PV: Portal Vein
ICE: Intracardiac Echo
IVUS: Intravascular Ultrasound
