# Supplementary figures and images for: Successful direct intrahepatic portosystemic shunt (DIPS) creation following transmesenteric porta hepatis access in a young patient with recurrent variceal bleeding
Source: CVIR Endovasc. 2023 Dec 19;6:63. doi: 10.1186/s42155-023-00377-8 (PMC10730482; doi:10.1186/s42155-023-00377-8)

## Slide 1
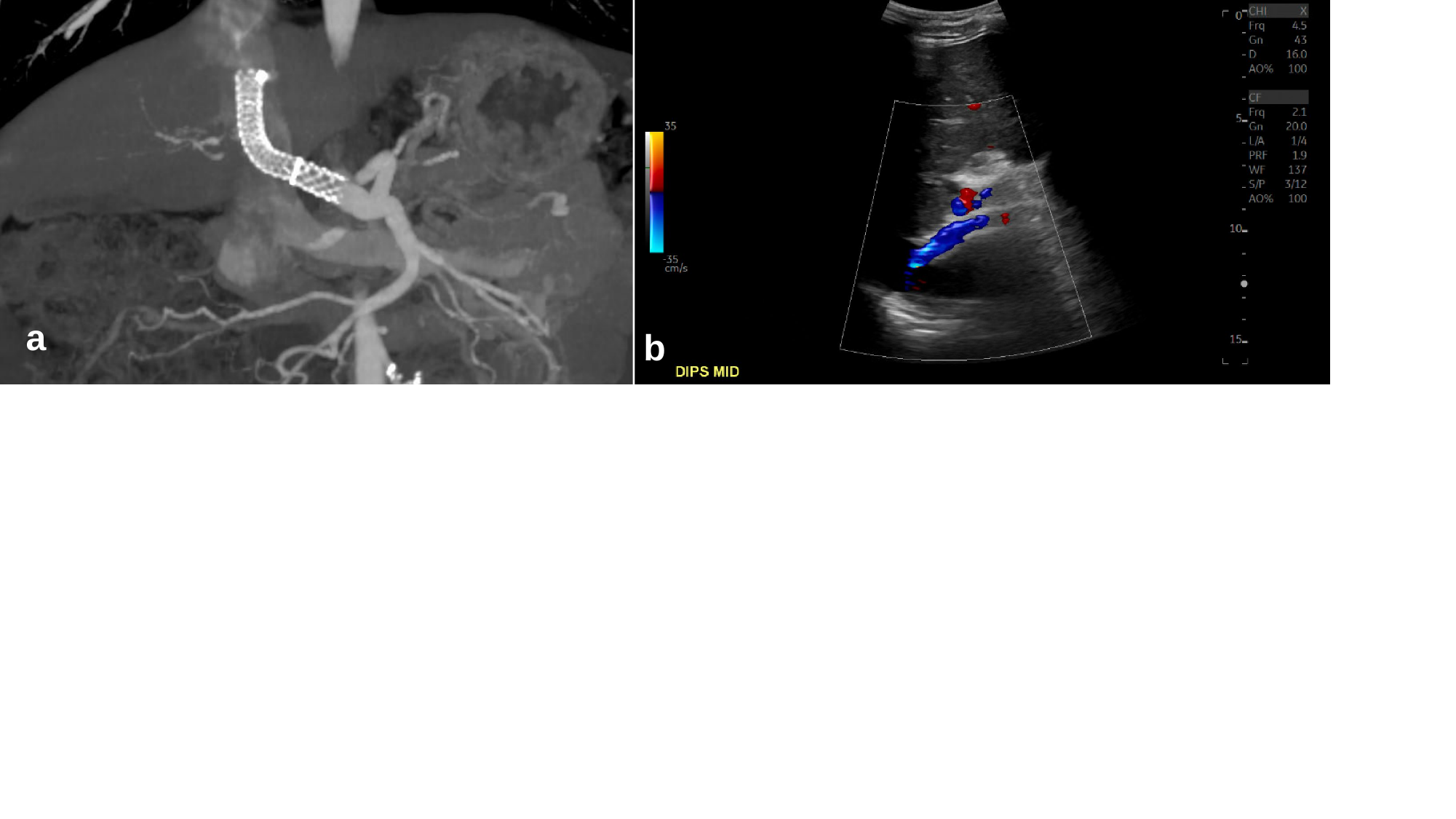

a
b

Supplement: Supplementary file 1 — Additional file 1: Supplemental Figure 1. A coronal image from multiplanar reformatted maximum intensity projections created from a CT angiogram performed 1-month post-procedure (a) demonstrates patency of DIPS stent and a subsequent (b) US performed 2-months post procedure demonstrate stent patency. [file 42155_2023_377_MOESM1_ESM.pptx]
